# The Feasibility and Acceptability of Using a Digital Conversational Agent (Chatbot) for Delivering Parenting Interventions: Systematic Review

**DOI:** 10.2196/55726

**Published:** 2024-10-07

**Authors:** Max C Klapow, Andrew Rosenblatt, Jamie Lachman, Frances Gardner

**Affiliations:** 1 Department of Experimental Psychology University of Oxford Oxford United Kingdom; 2 Department of Social Policy and Intervention University of Oxford Oxford United Kingdom; 3 Centre for Social Science Research University of Cape Town Cape Town South Africa

**Keywords:** chatbot, parenting intervention, feasibility, acceptability, systematic review, implementation

## Abstract

**Background:**

Parenting interventions are crucial for promoting family well-being, reducing violence against children, and improving child development outcomes; however, scaling these programs remains a challenge. Prior reviews have characterized the feasibility, acceptability, and effectiveness of other more robust forms of digital parenting interventions (eg, via the web, mobile apps, and videoconferencing). Recently, chatbot technology has emerged as a possible mode for adapting and delivering parenting programs to larger populations (eg, Parenting for Lifelong Health, Incredible Years, and Triple P Parenting).

**Objective:**

This study aims to review the evidence of using chatbots to deliver parenting interventions and assess the feasibility of implementation, acceptability of these interventions, and preliminary outcomes.

**Methods:**

This review conducted a comprehensive search of databases, including Web of Science, MEDLINE, Scopus, ProQuest, and Cochrane Central Register of Controlled Trials. Cochrane Handbook for Systematic Review of Interventions and PRISMA (Preferred Reporting Items for Systematic Reviews and Meta-Analyses) guidelines were used to conduct the search. Eligible studies targeted parents of children aged 0 to 18 years; used chatbots via digital platforms, such as the internet, mobile apps, or SMS text messaging; and targeted improving family well-being through parenting. Implementation measures, acceptability, and any reported preliminary measures of effectiveness were included.

**Results:**

Of the 1766 initial results, 10 studies met the inclusion criteria. The included studies, primarily conducted in high-income countries (8/10, 80%), demonstrated a high mean retention rate (72.8%) and reported high acceptability (10/10, 100%). However, significant heterogeneity in interventions, measurement methods, and study quality necessitate cautious interpretation. Reporting bias, lack of clarity in the operationalization of engagement measures, and platform limitations were identified as limiting factors in interpreting findings.

**Conclusions:**

This is the first study to review the implementation feasibility and acceptability of chatbots for delivering parenting programs. While preliminary evidence suggests that chatbots can be used to deliver parenting programs, further research, standardization of reporting, and scaling up of effectiveness testing are critical to harness the full benefits of chatbots for promoting family well-being.

## Introduction

### Background

Parenting, even in ideal conditions, is a stressful and challenging experience that can manifest in a variety of ways, such as emotional distance from the child, exhaustion in the parental role, decrease in self-efficacy, and loss of a sense of accomplishment as a parent [1]. Parental mental health issues can significantly impact the behavioral outcomes of children, particularly depression and anxiety [2,3]. Thus, finding cost-efficient and scalable approaches to improve parenting skills, reduce parental stress, and support healthy child development is critical to promoting family well-being. In low- and middle-income countries (LMICs), the effects of poverty are exacerbated by existing public health emergencies such as humanitarian crises, displacement, and poor mental health care [4]. These emergencies are associated with increases in violence against children which, in turn, is associated with poor outcomes such as behavioral problems, intimate partner violence, and low cognitive stimulation [4]. Preventing and reducing child maltreatment and its negative developmental outcomes are also linked to the United Nations Sustainable Development Goals (eg, 16.2: “End abuse, exploitation, trafficking and all forms of violence against and torture of children” and 1.3: “Implement nationally appropriate social protection systems and measures for all, including floors, and by 2030 achieve substantial coverage of the poor and the vulnerable”) [5]. Global emergencies such as pandemics, climate change–related natural disasters, and conflict-related displacement have only further highlighted the increasing need to provide support to families coping with stress and promoting a positive child-parent relationship.

### Parenting Programs

Parenting programs (also “parenting skills training”) are interventions that aim to improve parenting skills and support parents in acquiring knowledge and or skills to improve the health and well-being of children, including improving the parent-child relationship [6]. These programs, often conducted in group settings, can have a range of theoretical underpinnings and are typically manualized. They are flexible in length, typically ranging 8 to 12 weeks, and can be delivered in a variety of community settings by trained facilitators or subject matter experts [7]. Delivery components typically include (1) presentation of new information (eg, a framework for communicating with the child during an argument), (2) introduction of exercises and opportunities for guided practice (eg, structured scenarios with role-playing), (3) facilitated group discussion, (4) home assignments to apply learned skills with children, and (5) opportunities to provide feedback and discuss home assignments [8].

Programs can be designed for parents individually, as couples (if applicable), with children or adolescents present, or without. Typically, these programs aim to achieve a combination of (1) *educating* parents by providing new information, (2) shifting *attitudes* about parenting practices, and (3) changing the *behavior* of parents [9].

There is extensive evidence to suggest that parenting programs can increase positive parenting skills, improve the parent-child relationship, reduce the use of harsh discipline, and improve child behavioral problems [5]. Programs have been specifically designed for resource-limited settings [10,11], and some can be effectively integrated with other public initiatives such as cash transfer programs [12]. Programs such as Incredible Years, Triple P, Parent-Child Interaction Therapy, Parent Management Training Oregon, Strengthening Families, and Parenting for Lifelong Health have demonstrated to have shown positive outcomes and, in some cases, long-lasting effects [5]. The effectiveness of parenting programs has led to international promotion and scale-up with the support of organizations such as the United Nations International Children’s Emergency Fund and the World Health Organization [13]. Effective scale-up of parenting programs may also thus create a delivery pipeline for other related interventions, such as parental or child-specific mental health interventions, gender-based violence reduction interventions, or integration with other public health initiatives.

### Digital Behavior Change Interventions

*Digital behavior change interventions* (DBCIs), also referred to as behavioral intervention technology-based interventions, are interventions that use technology to support and promote healthy behaviors [14]. These may include interventions supported or delivered via a range of technologies such as websites, mobile apps, software, sensors, or hardware devices to change emotions, behaviors, or cognitions [15,16]. DBCIs can be used to increase the reach of in-person social interventions, particularly to populations that lack access to in-person programs or where in-person services are unavailable. DBCIs can be *guided*, which includes a significant in-person, synchronous component to support implementation, such as an internet-based program for reducing anxiety, which also includes regular low-touch support from a therapist or peer support [17]. They can also be *self-guided*, in which the intervention is administered completely digitally and can be completed asynchronously, similar to a manualized workbook-driven intervention [18,19]. DBCIs are often used in health settings [20-22].

### Chatbots

*Digital conversational agents*, or “chatbots,” are a type of self-guided DBCI. Chatbots respond to written and spoken language with text or spoken language, which can be prewritten or generated by artificial intelligence. Their capability is far-ranging; the simplest implementation of chatbots uses predefined algorithms where specific outputs are triggered by specific inputs from the user, while a highly sophisticated chatbot may use an artificial intelligence model to generate novel responses and learn from a user’s behavior to personalize responses over time [23]. Chatbots can be particularly useful for emulating human interaction and have been used successfully in physical health care, mental health care, and educational settings. In some cases, chatbots have demonstrated levels of trust with study participants similar to in-person interventions with physicians, therapists, or educators [19,24,25]. Chatbots can also be combined with other intervention modalities to support sustained engagement or on-demand, interactive access to intervention content [26,27].

Chatbot-based implementations of parenting programs can be delivered via internet-based messaging platforms (eg, Facebook Messenger, WhatsApp, and Signal); mobile apps that embed the chatbot within; and SMS text messaging, which does not require a mobile internet connection, is capable of sending multimedia content, and can be accessed at any time. SMS text messaging–based support messages have already been used to support in-person parenting programs in LMICs [28]. Their automated and highly customizable design makes the mode of delivery potentially useful for intervention settings that lack access to in-person services, require flexibility in participating in an intervention (such as a parenting program), or prefer a lower-intensity form of intervention. SMS text messaging delivery also has cost implications for providers, making them less feasible for wide-scale use in low-resource settings without government or telecom provider partnerships. With the introduction of powerful large language models capable of replicating highly accurate syntax and tone, there is a newfound need to understand the extent to which chatbot technology can be a suitable method for delivering interventions to populations experiencing barriers to in-person implementations.

Past reviews have examined the feasibility, acceptability, intervention characteristics, and effectiveness of digital parenting interventions, particularly for infants and young children [29,30]. These reviews have focused primarily on more complex digital modalities that include internet-based multimedia content, digitally supported interventions with primary in-person components, and technology that connects parents with in-person support [31,32]. Little work has focused on self-guided digital interventions such as chatbots. Preliminary pilots and trials of parenting programs delivered via chatbots have begun to be published, though, to the best of our knowledge, no synthesis has examined whether the evidence indicates that chatbots are a feasible and acceptable method for delivering parenting programs. Answering this question is critical for guiding future research in scaling up chatbot-based parenting programs. It is essential to evaluate the feasibility and acceptability of chatbot-based parenting programs as a whole, rather than focusing solely on individual studies. Understanding these aspects is crucial for determining the viability of this technology as a route for intervention delivery as well as developing it further. The aim of this study is to systematically review the existing studies reporting on the feasibility and acceptability of chatbot-delivered parenting interventions. We aim to describe the various types of parenting chatbots, explore the methods used to assess the feasibility and acceptability of chatbot-based parenting interventions, and evaluate the quality of evidence supporting this technology.

## Methods

### Reporting Guidelines

The design of this study followed the *Cochrane Handbook for Systematic Review of Interventions* [33] and the updated 2020 PRISMA (Preferred Reporting Items for Systematic Reviews and Meta-Analyses) guidelines for conducting and reporting systematic reviews [34].

### Inclusion Criteria

Studies were included if they targeted parents of children aged 0 to 18 years. The intervention needed to report an explicit focus on improving overall psychosocial well-being of family via advances in parenting, including reducing negative phenomena such as violence against children, abuse of children, and harsh parenting practices. The intervention needed to be delivered in the form an interactive conversational agent (“chatbot”) but could do so through any digital modality (internet based, mobile app, or SMS text messaging). For example, a website delivering a parenting skills training program to reduce child behavior problems and improve the parent-child relationship would only be included if the content was delivered via an identifiable, automated conversational agent within the website. Chatbots with and without artificial intelligence models for generating responses were included. In addition, the chatbot needed to be the primary component of intervention delivery, rather than as an add-on for monitoring or support purposes; studies with in-person components outside of onboarding were excluded. Intervention content could vary but needed to aim mainly and explicitly to improve parenting skills, including knowledge of or attitudes about parenting practices, self-care as it relates to parenting, the parent-child relationship, and preparing for parenting. Interventions that included lifestyle-related interventions were only included if the intervention content targeted changes in parental knowledge, attitudes, or behaviors. Studies in English and Spanish were included. No time restrictions were imposed on articles, though it was noted that studies before the 1990s would likely not meet the criteria, as this predated the internet. Data extracted from peer-reviewed published articles and gray literature, such as reports of ongoing studies, protocols, conference proceedings, and dissertations, were included to identify full reports of studies. Any study design meeting the abovementioned criteria was included to characterize this literature as broadly as possible.

### Exclusion Criteria

Solely qualitative articles were excluded. Studies with in-person components outside of onboarding were excluded. Studies that did not explicitly focus on improving overall psychosocial well-being of family via advances in parenting, including reducing negative phenomena such as violence against children, abuse of children, and harsh parenting practices, were excluded. Articles that did not feature an interactive conversational agent (“chatbot”) or were delivered via nondigital modalities were excluded. Studies where the chatbot was not the primary component of intervention delivery were also excluded. Articles in languages other than English and Spanish were excluded. Studies with no clear target on improving parenting skills, including knowledge of or attitudes about parenting practices, self-care as it relates to parenting, the parent-child relationship, and preparing for parenting, were not considered. Interventions that included lifestyle-related interventions but did not target changes in parental knowledge, attitudes, or behaviors were excluded.

### Primary and Secondary Outcomes

Primary outcomes of this review were measures of implementation, acceptability, and secondary measures of family well-being as measured by changes in parental knowledge, attitudes, behaviors, and psychological well-being (including symptoms of anxiety or depression) as well as child outcomes, such as reduction of behavioral or emotional problems. If multiple measures of implementation and acceptability were reported, these were categorized into primary and secondary measures with respect to their reporting within the study.

Due to the nascency of the literature, criteria for inclusion were developed to maximize *sensitivity* across population and outcome descriptors, while also maximizing *specificity* with the type of intervention. Nonrandomized studies, including feasibility and acceptability studies, as well as quasi-experimental studies, were included alongside randomized trials. Further details regarding the inclusion and exclusion criteria can be found in Textbox 1.

**Intervention inclusion and exclusion criteria.**

**Inclusion criteria**

- Intervention targets parents of children aged 0 to 18 years
- Intervention aims to improve the overall psychosocial well-being of family via changes in parenting, including reducing negative phenomena, such as violence against children
- Intervention is delivered via a digital, interactive conversational agent (“chatbot”)
- Intervention primarily and explicitly aims to improve parenting skills, including enhancing knowledge and attitudes

**Exclusion criteria**

- Intervention is delivered to children (but may have parental involvement)
- Intervention aims to improve outcomes tangentially related to well-being of family, including health reminders, disease prevention, weight management, and smoking cessation
- Intervention does not contain a digital, interactive conversational agent (websites, SMS text messages with no interactive component, and mobile apps with no interactive component)
- Intervention uses a digital chatbot as an add-on for monitoring or support purposes, rather than as a primary delivery mechanism
- Intervention delivers skills that are tangentially related to good parenting (child weight management, reducing unhealthy food intake, vaccine uptake, and health reminders) but are not parenting skills (mental health interventions)

### Search Strategy

The search was conducted in August 2023. Web of Science (Science Citation Index, Social Sciences Citation Index, Conference Proceedings Citation Index, and Emerging Sources Citation Index), MEDLINE, Scopus, ProQuest (Social Sciences Collection), and Cochrane Central Register of Controlled Trials were searched. All database searches were exported to Covidence systematic review software [35] for deduplication and screening. The search string was developed using the PICO framework (Population, Intervention, Comparator, Outcomes), shown in Textbox 1. A full search string can be found in Multimedia Appendix 1.

### Study Selection

All stages of the study process, including title and abstract screening, full-text review, data extraction, and quality assessment, were double-screened by MCK and AR. Screeners were blinded until the team met to resolve conflicts. Conflicts not resolved by consensus were advised on by the senior reviewer (FG). Study selection was conducted independently by the main coder (MCK) and a trained coder (AR) by title, abstract, and then full text. Intercoder reliability was maintained at each step of the screening process. The main coder opted to establish reliability at each stage independently to account for the range of considerations associated with each stage [36]. The main coder recruited and trained the second coder by jointly screening 25 (1.4%) of the 1766 included studies. Any questions about inclusion criteria were addressed before independent screening of titles and abstracts. Full-text screening involved joint training and screening of 10 studies. The main coder provided training on data extraction variables, and discussions followed independent coding of a small number of selected studies. Percent agreement was calculated at each stage by comparing agreements to selections. Successful training required ≥90% agreement, exceeding standard practice [36,37]. Any disagreements not resolved by discussion were settled by a third coder. Textbox 1 was used as a reference for screening, and the author and second coder met twice to resolve conflicts identified between screening. All excluded articles were labeled with a reason for exclusion.

### Data Extraction

Before extraction, separate articles were selected from the study by Vissenberg et al [38], a different but topically relevant review to practice applying the data extraction template to a similar group of studies. Due to the heterogeneous nature of study design and interventions, a meta-analysis synthesis was not possible. Instead, the features of interest included variable measures of feasibility and acceptability, type of delivery, and income level of intervention setting, and any measures of effectiveness were narratively synthesized.

Primary feasibility outcomes were operationalized as the included study’s main reported quantitative metric of engagement, which could vary between studies. Primary acceptability outcomes were operationalized as the included study’s main reported quantitative metric of acceptability, and if multiple measures were reported, measures of participants’ (1) overall appraisal of the intervention, (2) reported likelihood of using the intervention again, or (3) likelihood of recommending the intervention to someone else were considered primary measures. Secondary feasibility outcome measures were any additional quantitative measures of engagement. Secondary acceptability measures were any additional quantitative or qualitative variables related to participants’ experience with the intervention, or (2) or (3), if (1) was reported. Any effectiveness measures reported by each study was also extracted. Reported barriers and facilitators to use, either through free-response items on end point surveys or through participant feedback, were also extracted.

### Assessment of Study Quality and Risk of Bias

Studies that met the eligibility criteria were assessed for quality and relevance using the Weight of Evidence (WoE) framework [39]. Each study was scored across three criteria: (1) WoE A: general quality, (2) relevancy of study design to review question, and (3) relevancy of intervention design to review question, to produce (4) an overall WoE score. Each criterion was given a score of 1 (“Low”), 2 (“Moderate”), or 3 (“High”). Criteria (2) and (3) are prespecified in the study by Gough [39]. Full WoE assessment criteria can be found in Table 1. To assess study quality more objectively, the *Standard Quality Assessment Criteria for Evaluating Primary Research Papers from a Variety of Fields* (QualSyst) [40], which is designed for mixed methods, pre-post, and randomized designs, was used to score WoE A. Example items from QualSyst include the following: “Was the research question sufficiently described? (item 1),” “If interventional and blinding of subjects was possible, was it reported? (item 7),” and “Were the outcome measures well-defined and robust to measurement bias? (item 8).” A full list of items is provided in Multimedia Appendix 2.

**Table 1:** Weight of Evidence assessment rubric.

	Criterion A: QualSys^a^ quality appraisal tool score	Criterion B: relevancy of study design	Criterion C: relevancy of intervention design	Criterion D: averaged weight (criteria A, B, and C)
Low (=1.00)	0-0.55	Does not mention feasibility and/or acceptability AND/OR Makes conclusions about feasibility and acceptability without a clear link to evidence	<60% of the content delivered is parent skills training OR Partially automated, but manual components OR Has an equal number of components that are nondigital	1.00-1.75
Moderate (=2.00)	0.56-0.80	Mentions feasibility and acceptability measures AND/OR Measures are not adequate for assessing feasibility and acceptability; makes strong conclusions with mixed evidence	>60% of content is parent skills training OR Primarily automated but includes at least 1 manual component OR Mostly digital, may have some nondigital components	1.76-2.65
High (=3.00)	0.81-1.00	Explicitly reports feasibility and acceptability measures or effectiveness (if feasibility and acceptability has been established) AND/OR Measures are adequate, and conclusions about feasibility and acceptability are in line with the evidence provided	Only delivers parenting training (which may include parenting-specific stress management) AND Fully automated OR Fully interactive Completely digital	2.66-3.00

^a^QualSys: Standard Quality Assessment Criteria for Evaluating Primary Research Papers from a Variety of Fields.

Cut points from QualSyst were used to harmonize scoring between the 2 tools, where a QualSyst score of 0 to 0.55 was translated to a WoE score of 1 (“low”), 0.56 to 0.80 to 2 (“moderate”) and 0.81 to 1.00 to 3 (“high”).

**Figure 1:**
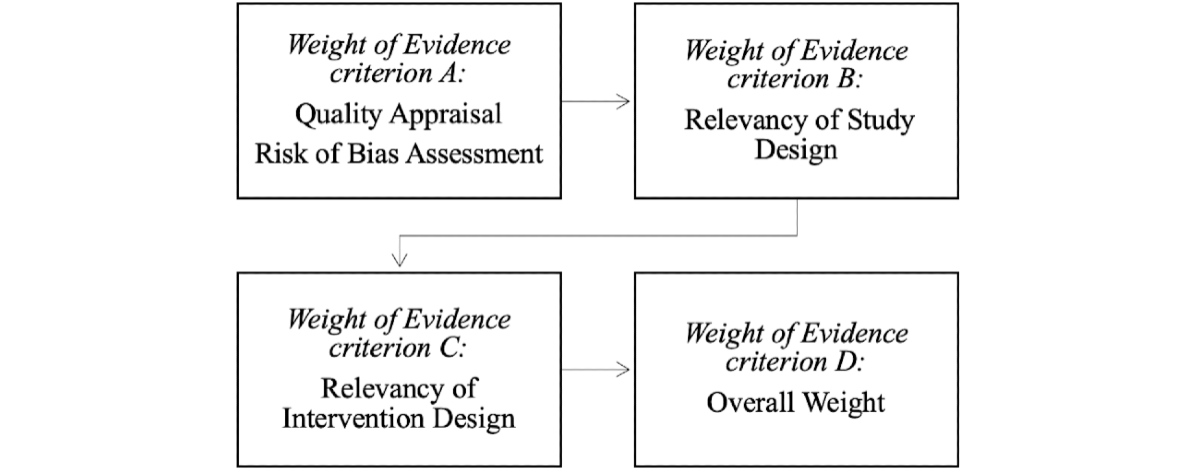
Quality and risk of bias assessment.

To assess risk of bias, domains from the Cochrane Risk of Bias in Nonrandomized Studies of Interventions and Risk of Bias Tool version 2 [41,42] were identified and assessed against the 14 criteria in the QualSyst tool. Descriptions of how to assess each domain in the Cochrane Risk of Bias in Nonrandomized Studies of Interventions were used to guide the review process. The quality and risk of bias assessment process is described in Figure 1.

## Results

### Included Studies

**Figure 2:**
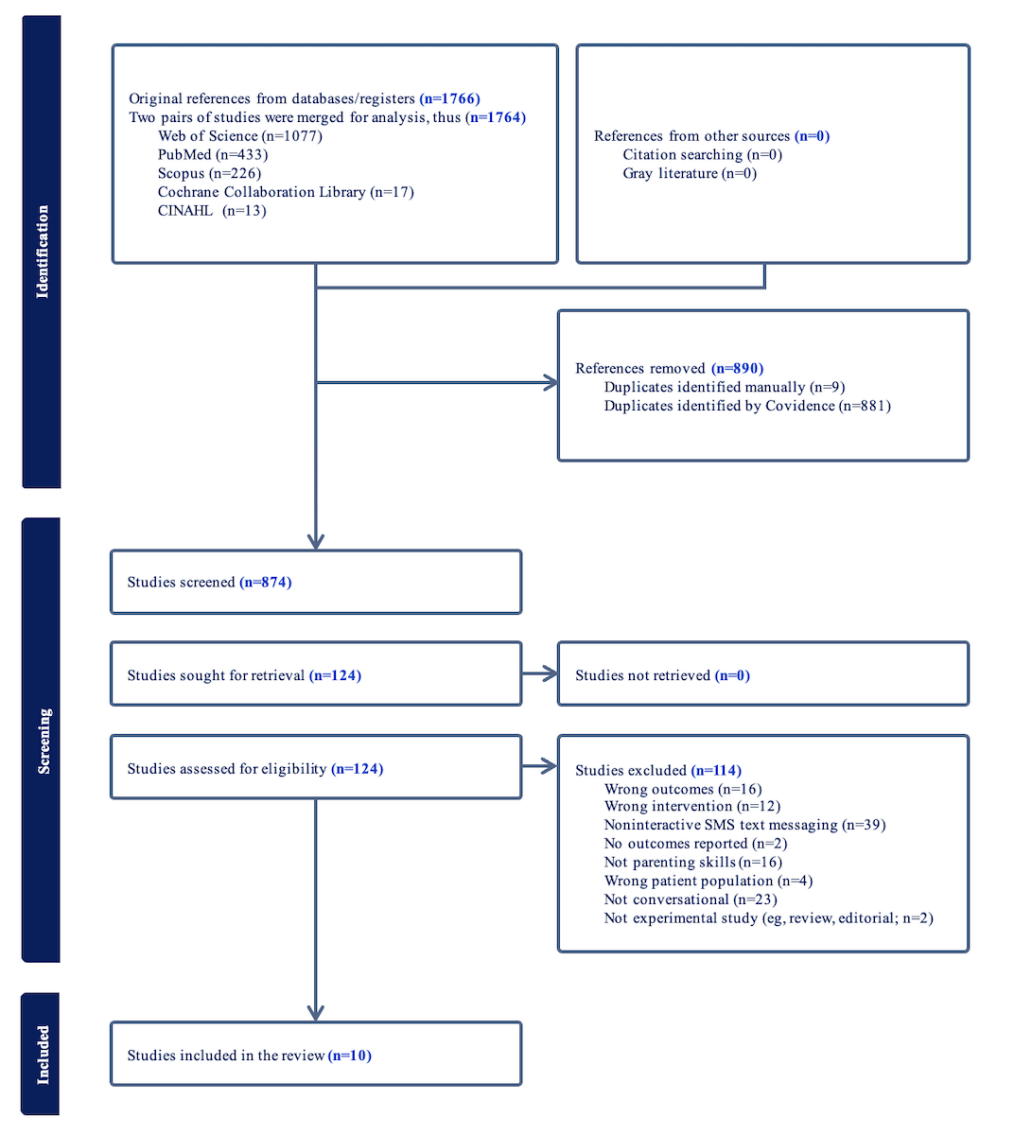
PRISMA (Preferred Reporting Items for Systematic Reviews and Meta-Analyses) study flow diagram.

The search yielded 1766 results, and 874 studies remained after deduplication (Figure 2). After title and abstract screening, full text of 124 studies were screened and 114 were excluded, leaving 10 included studies. The most common reasons for exclusion were the intervention being noninteractive (39/114, 34.2%); digital, but not in a conversational messaging format (23/114, 20.1%); did not include parenting-related outcomes (16/114, 14%); and did not deliver parenting skills as a primary component of the intervention (16/114, 14%). The complete list of exclusions can be seen in Figure 2. A total of 4 articles were merged into 2 studies: (1) the studies by Fletcher et al [43,44], due to the 2019 publication describing the development and intervention content and 2020 describing the feasibility study, and (2) the studies by Entenberg et al [45,46], as they report on different, relevant aspects of the same trial.

Among the 10 studies included, 8 (80%) were conducted in high-income countries—3 (30%) in Australia [43,44,47]; 2 (20%) in Argentina [46,48]; and 1 (10%) each in the United States [49], Taiwan [50], and Singapore [51]. A total of 2 studies were carried out in middle-income countries, with 1 in Brazil [52] and 1 in Peru [53]. The total participant pool across all studies (n=772) was drawn from diverse settings, including inpatient, outpatient, university, and community settings. The studies focused on parents with children spanning various age groups: 3 studies involved parents with infants aged 0 to 3 months [43,44,52], 4 studies targeted parents with children aged 2 to 11 years [46-48,50], 1 study addressed adolescents aged 13 to 18 years alongside the parenting intervention [49], another study included pregnant women and mothers with children aged 0 to 6 years [53], and 1 study focused on prospective parents, evaluating the intervention with men and women of childbearing age who mostly did not have children [51]. A full description of the characteristics of the included studies can be found in Table 2.

**Table 2:** Sample characteristics of the included studies.

Study, year	Country	Service setting	Income level	Study design	Population	Participant mean age	Method of recruitment	Total number of participants	Intervention length	Theoretical orientation (if stated)	Delivery type
Entenberg et al [45,46], 2023	Argentina	Not included	High-income setting	Randomized controlled trial	Parents in Argentina with at least 1 child aged 2 to 11 years	35.85 (SD 5.77)	Facebook posts and email list advertisements	170 (intervention group: 89; control group: 81)	15 minutes	Incredible Years Parenting Programme; behavior change techniques	Facebook Messenger
Fletcher et al [43], 2017	Australia	Not included	High-income setting	Pilot feasibility and acceptability study	Fathers expecting a child within 6 months or fathers with infants younger than 3 months	33.7 (range 21-59)	Advertisement posters in community centers, through Facebook forums, and at the hospital neonatal intensive care unit via trained staff	46	6 weeks	Psychoeducation, mood monitoring, and awareness	SMS text messaging
Fletcher et al [44], 2020	Australia	Not included	High-income setting	Pilot feasibility and acceptability study (no effectiveness)	Partners of mothers diagnosed with perinatal mental illness	29.3	Partners were invited after clinical interview at regional health centers	23	44 weeks	Psychoeducation, mood monitoring, and awareness	SMS text messaging
Mason et al [49], 2021	United States	Health care clinic	High-income setting	Randomized controlled trial	Parents of adolescents (aged 13 to 18 years) participating in the substance abuse prevention program	Parent mean age not reported	Community partner advertisement	52 (parents); 69 (adolescents)	4 weeks	Behavioral-skill framework (Dishion et al [54])	SMS text messaging
Entenberg et al [48], 2021	Argentina	Not included	High-income setting	Randomized controlled trial	Parents aged ≥18 with at least 1 child aged 2 to 10 years; not seeking psychological treatment	33.3% (n=11) of the participants were aged between 30 and 33 years, 30.3% (n=10) aged between 34 and 37 years, and 36.4% (n=12) were aged ≥38 years	Facebook posts	33	20 minutes	Incredible Years Parenting Programme	Facebook Messenger
Barreto et al [52], 2021	Brazil	Hospital	High-income setting	Intervention development and acceptability evaluation	New mothers aged >18 years with newborns of at least 24 hours old	24.4	Approached by research team in hospital and asked to participate	142	No time limit	Not stated	Within app
Downing et al [47], 2018	Australia	University (for initial onboarding)	High-income setting	Randomized controlled trial	Parents of children aged 2 to 4 years	Intervention group: 36.1 (3.9); control group: 34.1 (3.7)	Snowball method through community outreach and advertising	57 (Intervention group=30, control group =27)	6 weeks	Behavior change (CALO-RE)	Text messaging
Yu et al [50], 2023	Taiwan	Not reported	High-income setting	Intervention development and acceptability evaluation	Parents with childrearing difficulties	NR^a^	NR	58	12 weeks	Behavior change	Within app
Chua et al [51], 2023	Singapore	Tertiary public hospital	High-income setting	Intervention development and acceptability evaluation	Men and women of childbearing age; 10 with no children, single	26.7	Convenience sampling	11	28 weeks	Bandura self-efficacy theory, positive psychology, and psychoeducation	Within app
Jäggi et al [53], 2023	Peru	In home for onboarding and baseline interviews	Low-income setting	Pilot feasibility and acceptability study	Pregnant women and mothers with children aged 0 to 6 years	29	Convenience sampling	180	20 weeks	NR	Facebook Messenger

^a^NR: not reported.

### Study Design and Intervention Structure

A total of 3 studies were randomized, 4 were nonrandomized evaluations of intervention feasibility and acceptability, and 3 were intervention development reports that included preliminary surveys of acceptability. A full description of the characteristics of included studies can be found in Table 2. Participants were recruited by diverse methods, with advertisements on social media in parent groups being the most common. Sample sizes ranged from 11 to 170 participants. The 10 studies evaluated 8 distinct interventions delivered via SMS text messaging (4/10, 40%), Facebook Messenger (3/10, 30%), and a mobile app (3/10, 30%). While all the intervention aims included improving parenting skills, specific content varied and included positive praise, improving the parent-infant relationship, reducing parental stress, improving communication skills, and improving parental confidence. Intervention duration ranged from 15 minutes to 11 months, with 1 intervention [52] allowing parents to use the chatbot as long as needed with a prespecified end time to the pilot or experimental period. Theoretical orientation was not clearly reported in most cases, but behavior change (4/10, 40%) and psychoeducation (3/10, 30%) were the most reported.

### Factors Related to Implementation and Acceptability

#### Interactivity

Interactivity varied between interventions and was difficult to compare. One dimension of interactivity is the ability of the chatbot to respond realistically to queries or responses from participants. For example, the chatbot assessed by Entenberg et al [45,46,48] could be considered highly interactive, as they were supported by an artificial intelligence model that produced realistic speech-like text and could respond to participant messages that may have not been predicted by intervention developers. The other interventions were automated but used prewritten text message flows. As a result, intervention developers either predicted possible responses that the chatbot could respond to, or, more often, had specific response options embedded in messages to cue participants. Another dimension of interactivity is the extent to which content requires a response from participants. Generally, while some included studies gave examples of messages or templates, no studies had content flows accessible to independently assess the types of responses required from participants to interact with the content. Barreto et al [52], Entenberg et al [45,46,48], and Mason et al [49] delivered content that was both conversational and required complex textual responses to prompts from the chatbot, whereas Fletcher et al [43,44] and Downing et al [47] used templated messages that embedded cued responses to participants in messages and did not require complex textual inputs from the participant to continue. The latter studies also sent messages less frequently, and some messages did not require responses from participants. Considerable variation in both the degree of interactivity and the theoretical orientation of interventions, coupled with differences in their duration, poses a significant challenge in assessing the influence of interactivity on participant engagement.

#### Length of the Intervention

Intervention length also varied substantially, which can be attributed in part to variable approaches for the intended aim of the intervention for the participant. The studies by Barreto et al [52] and Fletcher et al [43], for example, were explicitly designed to serve as an on-demand source of information for parents to access or be prompted by over a long period, as evidenced by the substantially longer intervention period (note: the study by Barreto et al [52] does not specify a maximum length of intervention). In contrast, the intervention tested by Entenberg et al [45,46,48] was brief, lasting <30 minutes, and focused on a specific parenting skill. These interventions represent 2 extremes in terms of length within the review and demonstrate the relationship between purpose and duration. This relationship is also evident when examining the relative interactivity of the chatbot interventions. For instance, the intervention by Entenberg et al [45,46,48] involved a brief but highly detailed interactive exchange between the chatbot and the participant. In contrast, interventions by Mason et al [49] and Downing et al [47] were lighter touch, with messages requiring shorter responses that were often limited to “Yes,” “No,” or other affirmative responses.

#### Delivery Mode Informs Measurement Limitations

The considerable heterogeneity in measuring feasibility outcomes, such as retention, engagement, and completion, can in part be attributed to the platforms the chatbots were delivered on. For example, Barreto et al [52] delivered the chatbot in a downloadable mobile app, where it was possible to measure engagement characteristics such as mean length of engagement, which menus were accessed, and which information was accessed. Alternatively, interventions delivered via SMS text messaging, where that level of use data is not available, primarily measured engagement characteristics by number of responses or engagement with external links. Interventions delivered via Facebook Messenger reported less engagement-related data as via mobile app, but more than SMS text messaging–based interventions. Thus, a relationship between *how* the intervention is delivered and *what* engagement data can be collected exists and can affect feasibility reporting.

### Quality Assessment

#### Overview

The 10 studies included in the review were evaluated across three criteria: (1) quality of study and risk of bias, (2) relevancy of study design to review question, (3) relevancy of intervention to review question, using a WoE framework (refer to Table 1 and Multimedia Appendix 2 for evaluation criteria). A full list of quality assessment ratings may be found in Multimedia Appendix 3.

#### Individual Quality and Risk of Bias

Less than of the included studies were rated as high quality with a low overall risk of bias (4/10, 40%) or moderate quality with a low-to-moderate overall risk of bias (1/10, 10%), and half were rated as low quality with a high potential risk of bias (5/10, 50%). The most common reasons for lower ratings included unclear outcome measures, a lack of control for potential confounding variables, or unclear or inadequate analysis.

#### Relevancy of the Study Design

Most studies had highly relevant study designs (6/10, 60%). The most common reasons for studies being rated as “low” in relevancy of study design was due to not primarily measuring feasibility and for making conclusions about feasibility or acceptability without clear links to reported evidence.

#### Relevancy of the Intervention

Most studies had moderate (6/10, 60%) or highly relevant interventions (3/10, 30%). The most common reason for lower ratings were additional content unrelated to parenting skills rather than core content being unrelated to parenting skills.

#### Overall WoE

In total, 30% (3/10) of the studies demonstrated “high” quality, indicating robust methodology and high relevance to the research question. Of the 10 studies, 3 (30%) were rated as “low” quality, indicating issues with reporting and measurement. For example, Barreto et al [52] measured participant engagement by the mean number of access events but failed to clarify how this was operationalized or how confounding factors such as repeated access within a short period were controlled in the study. Overall, the evidence was moderately weighted (mean 2.36, SD 0.65), with 3 (30%) of the 10 studies receiving a high-weighted evidence rating, 4 (40%) studies receiving a moderate-weighted evidence rating, and 3 (30%) studies receiving a low-weighted evidence rating. This indicates that the current evidence moderately supports the feasibility and acceptability of chatbot-delivered parenting programs, but substantial development in both the evidence and reporting of findings is needed.

### Feasibility and Acceptability

#### Primary Implementation Measures

Retention was the most reported primary measure of implementation (8/10, 80%), though the operationalization of the measure varied between the 8 studies. A full description of the feasibility, acceptability, and preliminary outcomes can be found in Table 3. One study [52] reported the mean number of times the chatbot was accessed as a primary implementation measure, and 1 study [51] did not report any implementation measures. A total of 3 studies measured retention by participants fully completing the program, 2 studies [43,44] measured retention by participants who did not opt out of the intervention by the end of the evaluation period, 2 studies [47,50] measured retention by the number of participants who completed the postintervention survey, and 1 study measured retention by the number of active users at the end of the prespecified intervention evaluation period [53]. A weighted mean retention rate was calculated as 72.8% retention across studies, though this reflects retention rates reported by studies pooled together, without adjusting to compare similar measures to one another.

**Table 3:** Feasibility, acceptability, and effectiveness outcomes of the included studies.

Study, year	Total number of participants	Primary feasibility measure	Score (primary feasibility)	Secondary feasibility measure	Score (secondary feasibility)	Primary acceptability measure	Score (primary acceptability measure)	Secondary acceptability measures	Score (secondary acceptability)	Effectiveness outcome measure	Score (effectiveness)	Recommendation for use
Entenberg et al [45,46], 2023	170 (Intervention group: 89, control group: 81)	Retention	Dropout: 29% (26); completed intervention: 66% (59); completed follow-up: 28% (25)	Completion, dropout by skill and number of messages	Intervention group: 66.3% (59/81), skill 1: 17.98% (16), skill 2: 6.86% (5), skill 3: 7.35% (5), skill 4: 1.58% (1), and skill 5: 4.83% (3); number of messages: 49.8 (SD 1.53; range 20-80)	Satisfaction (1-5); Net Promoter Score (1-5)	Satisfaction: 4.19 (0.79); Net Promoter Score: 4.63 (0.66)	Survey (Likert 1-5): ease of use, comfort, absence of technical problems, interactivity, and usefulness in everyday life	Survey (Likert 1-5): ease of use: 4.66 (0.73) comfort: 4.76 (0.46) absence of technical problems: 4.69 (0.59) interactivity: 4.51 (0.77) usefulness in everyday life: 4.75 (0.54)	Self-efficacy, disruptive behavior	Mean 0.21 (SD 0.59); mean 0.37 (SD 0.96)	Recommended
Fletcher et al [43], 2017	46	Retention (*measured by number of* *participants who did not explicitly exit the intervention)*	87%	Accessing embedded links	Embedded links: most frequently clicked= 14/65 (22%); mood tracker: 24 (52%) responded ≥1 times	Recommend to others (Likert 1-5)	4.6	Structured phone interview (11 Likert scale questions): usefulness of intervention	4.32 (0.58)	NR^a^	NR	Recommended
Fletcher et al [44], 2020	23	Retention *(measured by use of embedded links and responses to the mood tracker)*	95.6% (22/23)	Embedded links: most frequently clicked= 8/23 (34.8%); mood tracker link, no response: 6/23 (26.1%)	1 (4.3%)	Likert Survey: “The messages helped me to develop a strong relationship with my new child.”	80%—agree or strongly agree	Likert survey: “The mood tracker messages, where I could respond to questions about how I was feeling, were useful for me”	43.8% (7)—agree or strongly agree	NR	NR	Recommended
Mason et al [49], 2021	52 (parents); 69 (adolescents)	Retention *(measured by number of participants who completed the intervention)*	98%	Response rate	93%	Helpfulness (measured at postintervention survey)	78%	Self-report: (1) satisfaction with no of texts and (2) use of skills	(1) 96% and (2) 91%	Parenting Practices Scale	0.34, SE 0.27, *P*=.21	Recommended
Entenberg et al [48], 2021	33	Retention *(measured by number of participants who completed the intervention)*	78.8% (26)	Number of messages sent	54.24 (SD 13.05)	Net Promoter Score (1-10)	7.44 (SD 2.31)	NR	NR	NR	NR	Recommended
Barreto et al [52], 2021	142	Mean number of times accessing chatbot	2	Length of conversation	27 seconds	Likert survey of experience and attitudes: “I liked using the GCBMB.”	96.4% “Totally agree” (137)	NR	NR	NR	NR	Recommended
Downing et al [47], 2018	57 (intervention group: 30, control group: 27)	Retention *(measured by number of participants who completed the intervention)*	Intervention group: 63%, control group: 70%	Number of replies to goal monitoring messages	83.3% (145/173)	Self-report use	95% (19/20) report reading at least 9 of 12 messages	NR	NR	Children’s sitting time (activPAL)	−30.6 minute/day	Recommended
Yu et al [50], 2023	58	Retention *(measured by the completion rate of the postintervention survey)*	51.7%	NR	NR	Chatbot usefulness for problem-solving *(Self-report questionnaire)*	>4.5/5 on all 6 items	NR	NR	NR	NR	Recommended
Chua et al [51], 2023	11	NR	NR	NR	NR	User acceptability testing survey, *(items 4-9; 1-7 Likert scale)*	Language appropriateness: mean=6.25; perceived friendliness: mean=5.9; enjoyability of use: mean=5.7	NR	NR	NR	NR	Recommended
Jäggi et al, [53], 2023	180	Retention *(measured by the number of active users at the end of the intervention period)*	41.7%	Intervention connectivity coverage	Urban (100%, 5/5), rural (22%, 10/44)	Chatbot usefulness (*Likert-like scale)*	87% rated “useful” to “very useful”; mean 4.37/5 (SD 1.00)	NR	NR	NR	NR	Recommended

^a^NR: not reported.

#### Secondary Implementation Measures

A total of 7 studies reported secondary measures of implementation. Fletcher et al [43,44] reported engagement as measured by the number of participants who accessed embedded links within the chatbot’s mood tracker at least once (24/46, 52% and 8/23, 26%, respectively). In addition to overall retention, Entenberg et al [45,46] assessed retention by intervention component, reporting a 79% (26/33) retention rate after the first of 5 components, as well as the number of messages sent between the chatbot and participant (mean 49.8, SD 1.53). Entenberg et al [48] also reported the number of messages sent (mean 54.24, SD 13.05). Similarly, Barreto et al [52] measured mean duration of chatbot-participant interaction (27.0 seconds). Mason et al [49] measured engagement by the percentage of participants who responded to the 3-month follow-up survey (48/52, 92%). Jäggi et al [53] was the only study that reported a non–engagement-related secondary measure of implementation examining intervention connectivity coverage for the chatbot across 49 test sites (urban: 5/5, 100%; rural: 10/44, 22%).

#### Primary Acceptability Measures

All 10 studies used self-report data to assess acceptability. The most common measure was a Likert-like scale with an item asking participants to indicate their overall attitudes toward the chatbot. Items varied in focus. Fletcher et al [44] asked participants to indicate the extent to which they agreed that “The messages helped me to develop a strong relationship with my child,” whereas Barreto et al [52] asked participants to rate the extent to which they agreed with the statement “I liked using the chatbot.” A total of 2 studies [46,48] assessed the likelihood of recommending the chatbot to a friend, as measured by the Net Promoter Score [55]. The study by Downing et al [47] was the only study that reported self-reported use as secondary measure of acceptability, as indicated by the percentage of participants reported reading at least 9 (95%) of 12 messages. While a weighted mean was not calculated due to the considerable heterogeneity in survey items, all studies reported high acceptability across their chosen measures.

#### Secondary Acceptability Measures

A total of 5 studies reported secondary measures of acceptability. All 5 studies [43,44,46,48,49] used quantitative self-report surveys to identify participant attitudes about ease of use, perceived usefulness, and comfort with the chatbot. Similar to primary acceptability and primary feasibility measures, there was considerable heterogeneity, though all studies reported high acceptability across additional measures. Entenberg et al [45,46] reported high ease of use (mean 4.66/5.0, SD 0.73), Fletcher et al [43,44] found high perceived usefulness (mean 4.32/5.0, SD 0.58; approximately 43.8% of participants agreed that the mood tracking interactive component was helpful), and Mason et al [49] found that 91% of the participants reported using skills learned from the chatbot within 3 months after the program.

### Preliminary Effectiveness

A total of 3 studies reported effectiveness outcomes. Entenberg et al [45,46] observed a small positive effect of the intervention on mean parental self-efficacy (Cohen *d*=0.36; mean 0.21, SD 0.59) and a moderate decrease in disruptive behavior (Cohen *d*=0.39; mean 0.37, SD 0.96), though neither reached statistical significance. Mason et al [49] also identified a small positive effect on parenting practices, measured by the Parenting Practices Scale (Gorman-Smith et al [56]; *F*_1,150_=0.57), but it did not achieve statistical significance (*P*=.45). Downing et al [47] did not report effectiveness outcomes related to parenting but focused on child sedentary behavior, a primary outcome related to the intervention aim. They found a significant positive effect of the intervention, indicating a decrease in the average number of minutes children spent sedentary per day (adjusted mean –22.3 min/day; 95% CI –80.8 to 36.3), suggesting preliminary effectiveness.

### Barriers and Facilitators to Use

A total of 5 studies reported on barriers and facilitators to use within the chatbot interventions [43,46-48,53]. All studies collected data through structured interviewing and Likert-like surveys. Parental busyness, impersonal and inflexible response from chatbots, technical problems, and repetitive or unengaging information were reported as barriers to use. Participants solely owning the device used for the intervention, technical support call buttons, encouraging messages, communication style and advice perceived as helpful, goal setting, and easy-to-understand messages were reported as facilitators for use. While other studies discussed potential barriers and facilitators, none reported formal methods for assessing these within the study.

## Discussion

### Principal Findings

This is the first study to review the implementation and acceptability characteristics of chatbot-delivered parenting interventions. Findings suggest that chatbots can be a feasible and acceptable method for delivery, but further research is required to assess whether engagement with the technology can be sustained as well as effectiveness compared to other digital parenting interventions. We identified an average retention rate of 72.8% across included studies. While all included studies individually conclude that chatbot interventions are implementable and acceptable, substantial development is needed in the standardization of definitions, measurements, and reporting. In addition, there is some evidence supporting moderate levels of implementation feasibility and acceptability of these interventions in high-income countries. However, there is limited evidence in middle-income countries and none in low-income countries. Implementation, primarily measured by retention, appeared to be high across included studies, as did retention as measured by program completion. Acceptability, primarily measured by self-report items about attitudes toward satisfaction and usefulness, was also considerably high in all included studies.

In addition, this review found that the delivery of parenting chatbots cited in included studies encountered external barriers such as parental busyness and internal barriers such as inflexible responses from the chatbots, technical problems, or repetitive information. Generally, chatbots were more acceptable when they used encouraging messages, easy-to-understand content, and content that was perceived as helpful or involved incremental goal setting. However, this review did not focus on identifying qualitatively reported barriers and facilitators to use, and no included studies looked specifically at these factors.

### Measuring Implementation and Acceptability in Digital Health Interventions

The high rates of retention and program completion reported from included studies on parenting program chatbots was unexpected given that digital health and mental health interventions generally suffer from low retention rates. While completely digital parenting programs have not been widely studied (refer to the study by Hansen et al [57], which reports retention rates of >70% for in-person interventions that are assisted by technology), a reasonable comparison to a parenting chatbot may be self-guided mental health mobile apps, as they are asynchronous, primarily or totally digital, and interactive. By contrast, Baumel et al [58] reported in a review of 93 mental health mobile apps that the median retention rate after 15 days was 3.9%. In a meta-analysis of 10 randomized controlled trials (n=1090) of digital self-guided interventions for depression, Karyotaki et al [59], found that 40% of participants dropped out before completing 25% of the intervention, and only 17% of the participants completed all the intervention. By contrast, this review reported that, across included studies, 72.8% of participants completed the intervention, which is higher than past reviews of digital health interventions have reported.

There are a few possible explanations for the high retention rates reported in this review. First, implementation and acceptability studies are particularly prone to publication bias, where researchers tend to publish studies with favorable outcomes for publication [60]. Second, compared to other types of digital health interventions that report lower retention, these interventions engaged parents with content primarily about the child and parent-child relationship, rather than solely the parent. This could be more compelling and not provoke the stress and subsequent avoidance associated with self-guided digital health and mental health interventions, which require internal motivation. Third, these interventions took place in high-income settings with onboarding and support from research teams for technical challenges, which could reduce attrition related to difficulty of use, stress, and lack of digital literacy. Finally, these high retention rates could indicate a more fundamental issue with measurements of engagement in digital interventions. Measuring engagement often includes retention, but retention can be measured differently depending on the study design and type of intervention. This lack of standardization in reporting guidelines can promote reporting bias in favor of statistics that indicate greater engagement. Standard measures of engagement, such as response or completion rate, could reduce heterogeneity in reporting. In this review, retention was operationalized variably across studies and did not necessarily align with standard definitions, opting instead for constructs such as program completion or end-survey completion. In some cases, program completion was indicated by not opting out of the chatbot, which may be alternatively described as program enrollment, rather than completion.

Operationalization of engagement also varied, leaving it subject to reporting bias. Some studies measured factors such as the number of interactions, length of responses, or number of modules completed. In contrast, studies limited to SMS text messaging could only track whether participants clicked on embedded links or responded to interactive messages. SMS text messaging–based interventions are also limited in how they track engagement. Links are commonly used to direct participants to a web browser page where engagement can be measured, given that SMS text messaging services lack the same depth of user data collection as mobile as. This highlights how the variability in intervention and study design can impact engagement and, as a result, reporting of retention. The ability to capture engagement data varies by platform; for example, a chatbot embedded in a mobile app can track engagement throughout the digital environment, whereas integrations with existing messaging platforms such as WhatsApp and Facebook Messenger can only collect engagement data as moderated by the platforms themselves. Current literature suggests using multiple valid measures of engagement to build a more complex, multidimensional model of engagement, though this is not always possible with SMS text messaging–delivered interventions. By contrast, trials of in-person parenting programs typically report higher retention rates, though these vary considerably due to barriers associated with in-person delivery [28,61].

### Limitations and Strengths

The review also had several limitations. First, the included studies were conducted exclusively in high-income settings, which severely limits the generalizability of these findings to LMICs. Digital literacy, access to consistent cellular service, access to private devices, and privacy concerns disproportionately affect populations in LMIC settings, which many of the included studies did not need to address. Second, the broad inclusion criteria contributed to the significant heterogeneity observed in the types of interventions studied, although some included interventions only marginally met the criteria. Third, the range in study quality may limit the generalizability of the study conclusions. Fourth, the heterogeneity of measurements and small sample size made conducting a meta-analysis impossible. The review also had several strengths. First, it is the first study to review chatbots as a mode for delivering programs that promote family well-being and searched a wide range of databases and gray literature comprehensively. Second, it uses a WoE approach to assess quality and risk of bias, which can more carefully account for study design, intervention design, and study quality when assessing the overall quality of evidence. Third, it compares studies’ approaches to measurement to identify how observed heterogeneity might impact reporting and interpretation of findings.

### Future Research

There are 3 primary areas of future research related to this study. First, future studies of chatbot-delivered parenting interventions should adopt and adhere to standardized reporting guidelines for digital health interventions such as the mobile health evidence reporting and assessment checklist [62]. Second, further development of guidelines that focus on standardized reporting of feasibility and acceptability measures will allow for between-study comparisons, which is critical for future reviews. Third, future studies should identify barriers to engagement more specifically within the digital environment through collecting additional use data as well as conducting qualitative interviews with participants.

### Conclusions

Digital conversational agents as a delivery mechanism for parenting interventions are still in the nascent stages. Significant development is needed in the measurement and reporting of feasibility and acceptability outcomes, as well as in identifying the barriers to and facilitators of engagement with these interventions. This study reviewed the evidence for the feasibility and acceptability of using digital conversational agents to deliver parenting interventions. Given the limited available evidence and its relevancy to the research question, the included studies suggest that digital conversational agents can be a feasible and acceptable way to deliver parenting interventions. A more detailed analysis revealed that considerable heterogeneity in the design of interventions and the measurement of feasibility and acceptability outcomes make comparing findings between studies more challenging and uncertain. However, the overall quality of the findings was moderate, and most of the evidence was in favor of demonstrating feasibility and acceptability. Importantly, these conclusions are drawn from limited evidence. This review highlights the need for more rigorous standardization of reporting on digital interventions, additional research designing and testing new parenting chatbot interventions, and scaling up effectiveness testing of the studies included in this review.

## Appendix

Multimedia Appendix 1 Systematic review search string.

Multimedia Appendix 2 Standard Quality Assessment Criteria for Evaluating Primary Research Papers from a Variety of Fields template.

Multimedia Appendix 3 Weight of Evidence ratings.

Multimedia Appendix 4 PRISMA Checklist.

## Abbreviations

DBCI: digital behavior change intervention
LMIC: low- and middle-income country
PRISMA: Preferred Reporting Items for Systematic Reviews and Meta-Analyses
QualSyst: Standard Quality Assessment Criteria for Evaluating Primary Research Papers from a Variety of Fields
WoE: Weight of Evidence
