# Supplementary material for: The Feasibility and Acceptability of Using a Digital Conversational Agent (Chatbot) for Delivering Parenting Interventions: Systematic Review
Source: JMIR Pediatr Parent. 2024 Oct 7;7:e55726. doi: 10.2196/55726 (PMC11494261; doi:10.2196/55726)
Supplement: Multimedia Appendix 1 [file pediatrics_v7i1e55726_app1.docx]

Appendix 1

*Database Search String*

| POPULATION | INTERVENTION | OUTCOME(S) |
| --- | --- | --- |
| Parent* or mother or maternal or father or paternal or caregiver or “care giver” or family | “conversational agent” or chatbot or “chat bot” or bot or “text message” or text-message or “SMS” or “digital tool” or “conversational interface” or “artificial intelligence chatbot” or “automated agent” or “chat agent” or “virtual coach” or “virtual tutor” AND Parenting or “parenting skills” or “positive parenting” or “parenting intervention” | child parent relation or child-rearing or “family functioning” or “family relation” or “family conflict” or “family life” or parent-child or maternal behaviour or paternal behaviour or “parent-child communication” or “parent education” or “parent training” or “child abuse” or “child neglect” or maltreatment or child violence |
