# Supplementary material for: The Feasibility and Acceptability of Using a Digital Conversational Agent (Chatbot) for Delivering Parenting Interventions: Systematic Review
Source: JMIR Pediatr Parent. 2024 Oct 7;7:e55726. doi: 10.2196/55726 (PMC11494261; doi:10.2196/55726)
Supplement: Multimedia Appendix 2 [file pediatrics_v7i1e55726_app2.docx]

**Appendix 2**

*Standard Quality Assessment Criteria for Evaluating Primary Research Papers from a Variety of Fields*

| **Study Name:** |  |  |  |  |  |  |
| --- | --- | --- | --- | --- | --- | --- |
| **Criteria** | **Yes** | **Partial** | **No** | **Not Applicable** | **Comments** | **Score** |
| Is the research question/study objective sufficiently described? |  |  |  |  |  |  |
| Is the study design evident and appropriate? |  |  |  |  |  |  |
| Is the method of subject or comparison group selection **or** source of input variables described and appropriate? |  |  |  |  |  |  |
| Is the subject and comparison group (if applicable) characteristics sufficiently described? |  |  |  |  |  |  |
| If intervention and randomization was possible, was it described? |  |  |  |  |  |  |
| If interventional and blinding of investigators was possible, was it reported? |  |  |  |  |  |  |
| If interventional and blinding of subjects was possible, was it reported? |  |  |  |  |  |  |
| Are the outcome and (if applicable) exposure measure(s) well defined and robust to measurement / misclassification bias? Are the means of assessment reported? |  |  |  |  |  |  |
| Is the sample size appropriate? |  |  |  |  |  |  |
| Are the analytic methods described justified and appropriate? |  |  |  |  |  |  |
| Is there some estimate of variance is reported for the main results? |  |  |  |  |  |  |
| Is there control for confounding? |  |  |  |  |  |  |
| Are results reported in sufficient detail? |  |  |  |  |  |  |
| Are conclusions supported by the results? |  |  |  |  |  |  |
| Total Score: |  |  |  |  |  | 0 |
